# Comparison of efficacy and safety of different tourniquet applications in total knee arthroplasty: a network meta-analysis of randomized controlled trials

**DOI:** 10.1080/07853890.2021.1991588

**Published:** 2021-11-02

**Authors:** Ziqin Cao, Jia Guo, Qiangxiang Li, Jianhuang Wu, Yajia Li

**Affiliations:** a Department of Spine Surgery and Orthopaedics, Xiangya Hospital, Central South University, Changsha, Hunan, China; b Department of Dermatology, Xiangya Hospital, Central South University, Changsha, Hunan, China; c National Clinical Research Center for Geriatric Disorders, Xiangya Hospital, Central South University, Changsha, Hunan, China; d Ningxia Geriatric Disease Clinical Research Center, People’s Hospital of Ningxia Hui Autonomous Region, Yinchuan, Ningxia Hui Autonomous Region, China; e National Clinical Research Center for Geriatric Disorders of Xiangya hospital, Central South University (Sub-center of Ningxia), Yinchuan, Ningxia Hui Autonomous Region, China; f Department of Hunan Institute of Geriatrics, Hunan People’s Hospital, Changsha, Hunan, China

**Keywords:** Knee replacement, tourniquet, tourniquet time, network meta-analysis, blood loss

## Abstract

**Objective:**

Total knee replacement (TKA) is an effective way to treat teratogenic and disabling knee diseases such as advanced osteoarthritis. Tourniquets are often used in TKA to reduce bleeding and to get a better visualization of the surgical field, while it is related to safety concerns. We did this network meta-analysis to comprehensively compare the efficacy and safety of various tourniquet application strategies.

**Method:**

PubMed, Embase, Cochrane Library, CNKI, and WanFang Database were systematically searched from January 1990 to May 2020. A network meta-analysis with a frequentist framework was done to assess the relative efficacy and safety by comparing seven clinical important endpoints.

**Results:**

38 eligible studies that assessed 3007 participants who underwent TKA were included in this network meta-analysis. Tourniquet inflation before osteotomy then deflation after wound closure effectively reduce perioperative bleeding (WMD compared with control group −234.66, 95% CI [–409.19 to −60.13]), while shortening the operation time (WMD −8.98, 95%CI [–14.07 to −3.88]) and reducing postoperative complications, including DVT (OR −0.58, 95%CI [–1.19 to 0.03]) and minor wound complications (OR −1.38, 95%CI [–3.00 to 0.25]). No difference was found in the late postoperative knee pain and function outcomes.

**Conclusions:**

Using tourniquets during the entire operation can effectively reduce blood loss, but it also can cause many safety problems, including DVTs, wound oozing, delayed healing, and serious wound complications. Tourniquet inflation before osteotomy then deflation after wound closure effectively can reduce perioperative bleeding while shortening the operation time and reducing postoperative complications, so it could be the ideal tourniquet application strategy in TKA.

**Key messages**
- This is the first study that comprehensively compared different tourniquet application strategies to evaluate their impact on postoperative recovery following TKA, and five clinically important endpoints were assessed in this study: perioperative blood loss, operation time, postoperative pain and function, and complications.
- We conclude that tourniquet inflation before osteotomy then deflation after wound closure could be the ideal tourniquet application strategy in TKA.

## 1. Introduction

Knee osteoarthritis (KOA) and rheumatoid arthritis (RA) are common knee diseases that cause joint pain and loss of function. Pharmacological treatments may be used early to improve symptoms, but for end-stage patients, total knee replacement (TKA) is often the only effective intervention [1,2].

Some studies have found significantly more intraoperative bleeding [3], which can further lead to the deterioration of surgical visualization, the extension of surgical time, and can interfere with the bonding effect of bone cement. To solve these difficulties, tourniquets are widely used during TKA. However, it was associated with a higher risk of complications, including Thigh pain and swelling, paresthaesia, vascular injury, and venous thromboembolism, subcutaneous fat necrosis, postoperative stiffness, poor wound healing, and delayed recovery of quadriceps strength [4–7].

Previous studies have confirmed that the prolonged application of a tourniquet is a key factor in complications that may be associated with prolonged ischaemic time for tissues [7–9]. More and more researchers have begun to minimize tourniquet time to determine whether the limited application of tourniquets in TKA can reduce complications and promote postoperative recovery.

Kvederas G et al. [10] compared the efficacy and safety of three tourniquet application strategies (Tourniquet inflation before incision then deflation after wound closure; inflation before incision then deflation after cement hardening; and inflation just during cementing). Their study demonstrated that tourniquet inflation before incision then deflation after cement hardening tends to give better outcomes. Inflation only during cementing was associated with the greatest total blood loss following TKA. Tai TW et al. [11] compared perioperative blood loss and postoperative pain between two groups with or without a tourniquet. They reported that the use of a tourniquet during TKA effectively reduces perioperative blood loss but was related to slightly more postoperative pain that did not affect recovery. A shorter duration of tourniquet application resulted in a faster recovery and less pain during the early postoperative period and a lower incidence of complications following TKA [12,13], but was associated with a significantly greater risk of blood loss and transfusion [14]. Therefore, the optimal timing of tourniquet application is still highly controversial and should be balanced with the increased blood loss and risk of transfusion with postoperative complications when using the tourniquet in TKA.

Until now, six different tourniquet applications have been proposed clinically, including non-use of a tourniquet (NTU); tourniquet inflation before incision than deflation after wound closure (throughout the operation, TTO); tourniquet inflation before incision and deflation after cement hardening (first half of operation, FHO); tourniquet inflation before osteotomy and deflation after wound closure (before osteotomy in operation, BOO); tourniquet inflation after osteotomy and deflation after wound closure (second half of operation, SHO); and tourniquet inflation only during cementing (middle of operation, MO).

Previously, numerous meta-analyses in this field have been published. For instance, Alcelik I et al. [15] conducted a meta-analysis of outcomes with and without a tourniquet in TKA, whereas Wang C et al. [16] presented a meta-analysis comparing tourniquet application only during cementation and long-duration tourniquet application in TKA. However, there still has been no systematic review evaluating the relative efficacy and safety of the various tourniquet application strategies or comparisons of these strategies to cases without tourniquet application in TKA. Accordingly, to comprehensively quantify the clinical efficacy and safety of different tourniquet applications and select the best strategy, we designed and conducted this network meta-analysis.

## 2. Method

### 2.1. Data Sources and searches

This network meta-analysis was designed and conducted based on the Preferred Reporting Items for Systematic Reviews and Meta-Analyses guidelines (PRISMA) [17].

PubMed, Embase, Cochrane Library, CNKI, and WanFang Database were systematically searched from January 1990 to May 2020, with the search terms ((“tourniquet” OR “tourniquet time” OR “tourniquet application” OR “tourniquet release”) OR (“ischaemic” OR “ischaemic injury” OR “tourniquet related ischaemic injury”) AND (“knee arthroplasty” OR “knee replacement” OR “TKA”)). Reference lists of relevant systematic reviews and identified articles also were reviewed to find additional eligible studies as completely as possible. No restriction was placed on the language of publication.

### 2.2. Study selection

Any article which met the following inclusion criteria were included: 1. The patients included in the study underwent total knee replacement or arthroplasty; 2. The study included a comparison among two or more different tourniquet release strategies; 3. The study used a randomized clinical trial design, whether blinded or not; 4. The study reported at least one desirable outcome: operation time, blood loss, functional rehabilitation, pain relief, the incidence of DVT, or other complications.

The exclusion criteria for this study were: 1. The study included another type of surgery, including unicompartmental knee arthroplasty or osteotomy; 2. Low-quality studies according to the Cochrane Handbook; 3. All animal studies and cadaver studies; 4. All reviews, systematic reviews and meta-analyses, conference abstracts, letters, and those without original study data.

### 2.3. Data extraction and quality assessment

Two authors reviewed the full manuscripts of all eligible studies and extracted relevant data from the studies, including author name, publication year, patient number, diagnosis, mean age, gender, surgical approach, whether to use tranexamic acid, whether to use drainage, whether to use anticoagulation and outcomes data. To avoid the influence of withdrawal bias, we preferred to extract data from the intention-to-treat analysis if available.

Two other authors conducted the methodological quality and bias assessment of studies using the Cochrane risk of the bias assessment tool [18]. The following indices were evaluated and ranked the as low risk of bias, unclear risk of bias, or high risk of bias: Sequence generation, allocation concealment, blinding, incomplete outcome data, selection outcome reporting, and other sources of bias. All disputes were resolved through discussion.

### 2.4. Outcome measures

Operation time, blood loss, postoperative pain, and function scale and safety endpoints were chosen as outcome measurements considered important for decision making. Blood loss included intraoperative, postoperative, and total blood loss, which were compared among groups to identify the efficacy of each tourniquet application strategy. The weighted mean difference (WMD) with 95% confidence intervals (CI) was used to measure the operation time and blood loss.

Early postoperative pain/function status (within one week after surgery) and late status (at least one month after surgery) were compared. No restriction was placed on the types of questionnaire used in pain evaluation. The function subscales of the Western Ontario and McMaster Universities Arthritis Index (WOMAC) were used to evaluate postoperative functional status. If the WOMAC function score was not measured or reported, the Lequesne Index or other functional measurement scales were used. The standardized mean difference (SMD) was used because results from different scales were included in the same network. Additionally, the range of knee flexion was compared to reflect the postoperative functional recovery.

Safety endpoints comprised DVT, minor and major wound complications. Minor wound complications included wound oozing, erythema, cellulitis, minor dehiscence, and superficial infection. Major wound complications included any condition that led to surgical failure, required a second surgery, or caused disability or death. The odds ratio [OR] with 95% confidence intervals (CI) was used to measure relative safety.

### 2.5. Statistical analysis

Traditional pairwise meta-analysis was conducted to compare the efficacy of the target drugs with placebo using RevMan (Review Manager. Version 5.3, Copenhagen, The Nordic Cochrane Centre, The Cochrane Collaboration, 2014). The heterogeneity across studies was tested by the Q and I2 statistics, in which *p* < .05 or *I*^2^ > 50% implies significant heterogeneity. A random-effects model was used if significant heterogeneity across studies was found, otherwise, a fixed-effects model was preferred.

The random-effects network meta-analysis was conducted using Stata/MP (version 14.0) in a frequentist method. The multivariate random-effects meta-regression was used to pool data with a proportional variance-covariance matrix, and a restricted maximum-likelihood method was used to assess model fit [19]. Global inconsistency tests and node-split tests were conducted, and the consistency model was adopted if no inconsistency was reported (*p*-value of *Z*-test >.05). If available, loop-specific inconsistency tests were also conducted to evaluate the inconsistency between direct and indirect comparisons within every closed triangle or quadratic loop. Inconsistency factors (IFs) and their 95% CI reported by loop-specific inconsistency tests reflect the consistency [20]. If inconsistency was reported in any network, the sensitivity analysis was used to identify the source of inconsistency and exclude these studies from this network. Funnel plots and Egger’s tests were conducted in Stata/MP to detect publication bias for each endpoint. The trim and filling method was used for further analyses to confirm whether significant publication bias was present [21]. The surface under the cumulative ranking (SUCRA) was used to rank the efficacy and safety of different drugs [22]. To select the most effective and safest drug simultaneously, cluster-ranking plots were constructed. Significant differences were considered between treatments when the corresponding 95% CI did not contain 1 for OR. *p* < .05 was considered statistically significant.

## 3. Result

### 3.1. Literature selection

**Figure 1.**
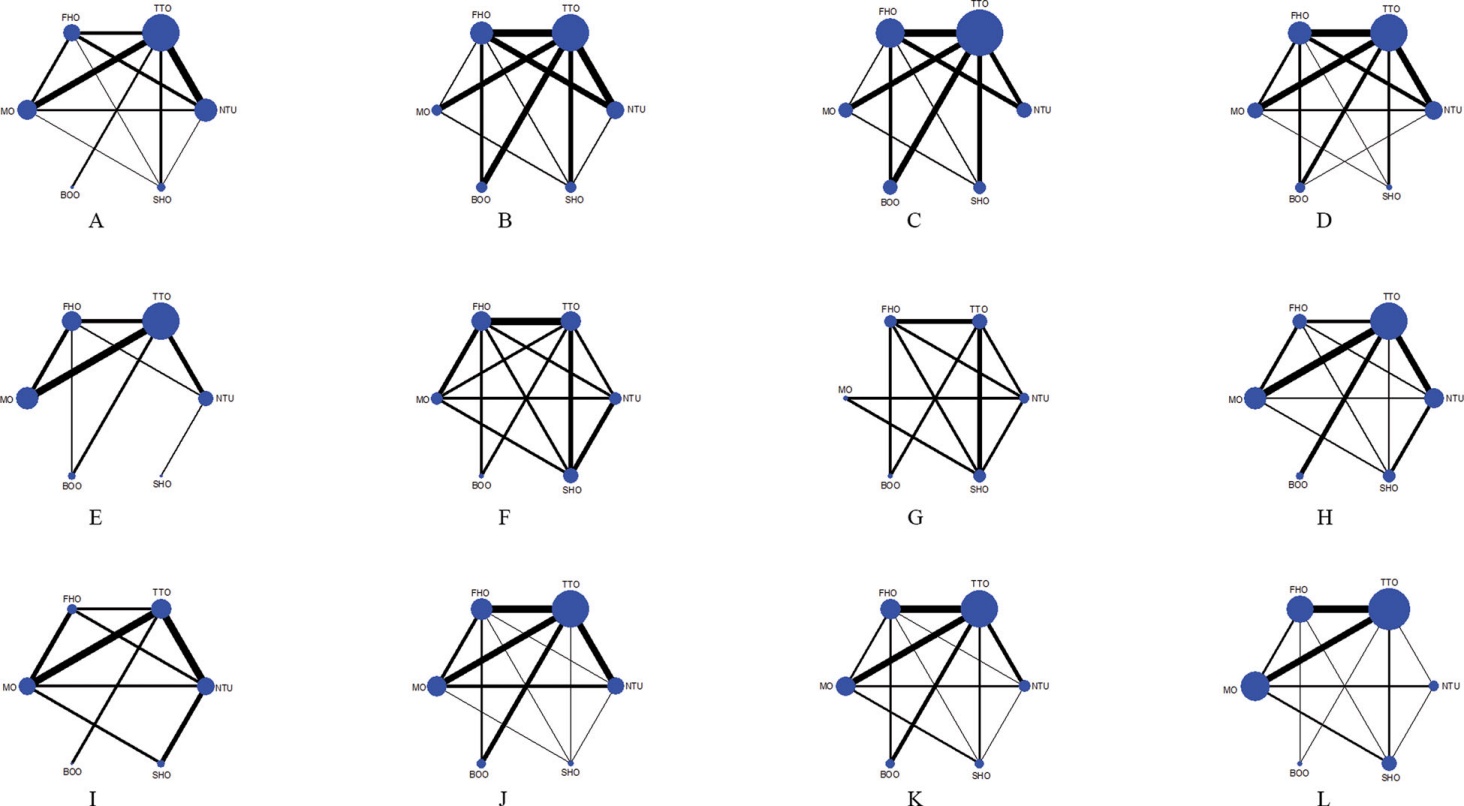
▪ ▪ ▪.

Thirty-eight eligible studies were identified [10–13, 23–56]. The PRISMA flow diagram is shown in Supplementary figure 1. Six different tourniquet strategies (NTU, TTO, FHO, MO, BOO, and SHO) were analyzed, and the NTU was selected as the standard control group. The network plot is presented in Figure 1.

### 3.2. Study characteristics

The mean age of the 3007 patients included in this study was 67.61 years (IQR 64.66 to 70.50 years) and the proportion of male patients was 31.79% (ranged from 10.91% to 53.23%) across studies. Most participants received primary TKA due to KOA or RA, except for five patients who underwent revision surgery. The medial parapatellar approach was the most common surgical approach (26 articles), followed by the mid-vastus approach (4 articles) and sub-vastus approach (1 article). Seven articles did not report the surgical approach used. Other baseline characteristics are presented in Supplementary Table 1. The details of quality and bias-risk assessments are presented in Supplementary Table 2. Based on these results, the main contributing factors to the risk of bias were performance bias, selection bias, and attrition bias.

**Figure 2.**
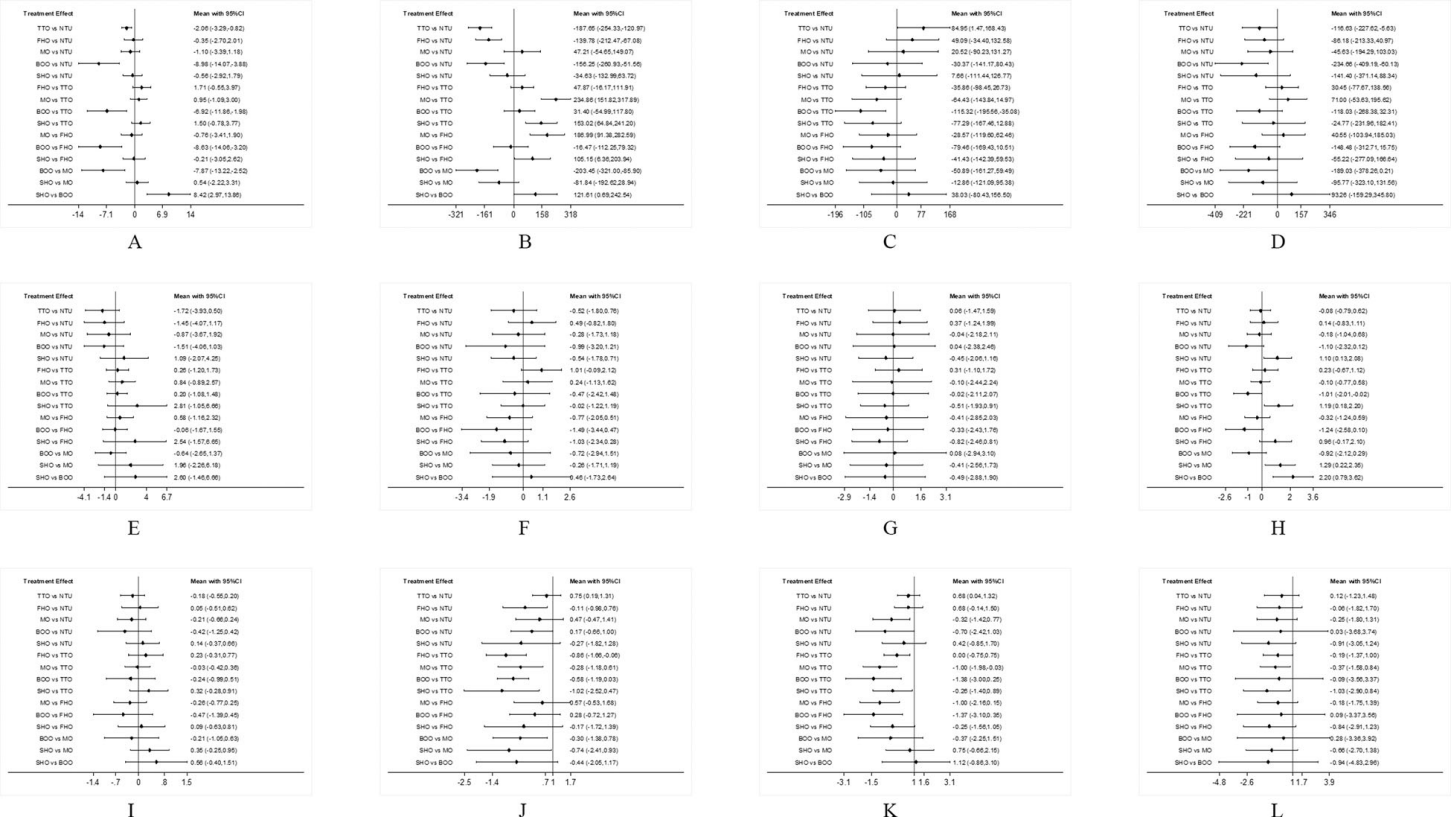
▪ ▪ ▪.

**Figure 3.**
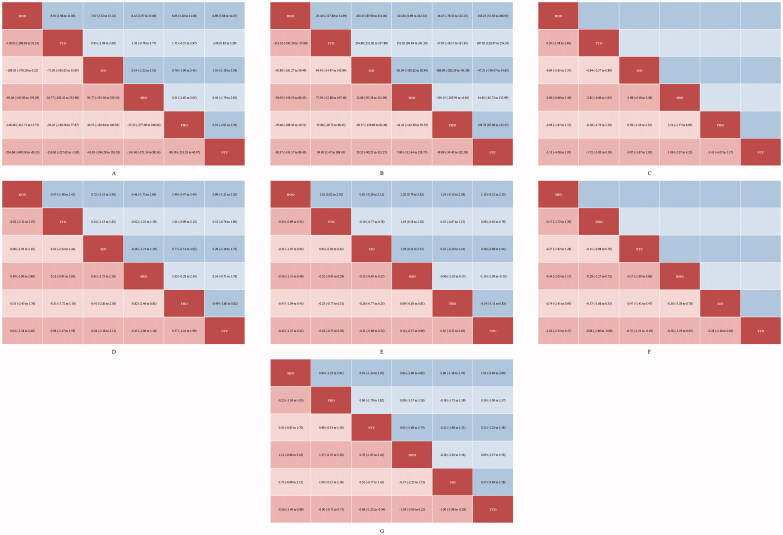
▪ ▪ ▪.

Publication bias was not reported in any network according to the results of funnel plots and Egger’s tests (Supplementary Figure 2). Results of cluster-rank analyses are presented in Supplementary Figure 3. The detailed results of conventional pair-wise meta-analyses are presented in Table 1. The forest plots of network meta-analyses are presented in Figure 2. The league plots which showed the relative effects between different groups are presented in Figure 3. Detailed SURCA values were presented in Supplementary Tables 3–5.

**Table 1.** The detailed results of conventional pair-wise meta-analyses.

Comparison (use vs. not use of tourniquet)	No. of trials	No. of patients	Diagnosis	Heterogeneity, *I*^2^	Effect index	Effect size
Operation time	17	1324	Osteoarthritis Rheumatoid arthritis Revision	79.90%	WMD with 95%CI	–3.991
						(–7.143 to −0.839)
Itraoperative blood loss	10	703	Osteoarthritis Rheumatoid arthritis	88.20%	WMD with 95%CI	–142.861
						(–175.053 to −110.669)
Postoperative blood loss	6	431	Osteoarthritis Rheumatoid arthritis	59.10%	WMD with 95%CI	81.281
						(35.233 to 127.329)
Total blood loss	15	1110	Osteoarthritis Rheumatoid arthritis Revision	92.20%	WMD with 95%CI	–79.279
						(–177.341 to 18.783)
Knee flexion range	6	676	Osteoarthritis Rheumatoid arthritis	39.30%	WMD with 95%CI	–0.785
						(–2.598 to 1.028)
Early postoperative function	5	452	Osteoarthritis Rheumatoid arthritis	71.60%	SMD with 95%CI	–0.304
						(–0.682 to 0.073)
Late postoperative function	4	288	Osteoarthritis Rheumatoid arthritis	0.00%	SMD with 95%CI	0.045
						(–0.186 to 0.277)
Early postoperative pain	8	788	Osteoarthritis Rheumatoid arthritis	97.30%	SMD with 95%CI	0.23
						(–0.733 to 1.194)
Late postoperative pain	7	716	Osteoarthritis Rheumatoid arthritis	73.40%	SMD with 95%CI	–0.036
						(–0.340 to 0.268)
Incidence of DVT	10	881	Osteoarthritis Rheumatoid arthritis Revision	0.00%	OR with 95%CI	1.394
						(0.879 to 2.209)
Minor complication	6	629	Osteoarthritis Rheumatoid arthritis	0.00%	OR with 95%CI	1.306
						(0.846 to 2.017)
Major complication	3	341	Osteoarthritis Rheumatoid arthritis	0	OR with 95%CI	0.945
						(0.251 to 3.555)

### 3.3. Operation time

#### 3.3.1. Conventional pair-wise meta-analysis

Significant heterogeneity of the included studies and interventions was reported (*I*^2^ = 79.9%), so the random-effects model was adopted. The use of a tourniquet overall was associated with a significantly shorter operation time than non-use (WMD −4.00, 95% CI [–7.14 to −0.84]).

#### 3.3.2. Network meta-analysis

A total of 33 articles were analyzed in this network. However, significant inconsistency was reported by the global inconsistency test (*p* < 0.05). In the node-split test and loop-specific inconsistency tests, inconsistency was found in the NTU – FHO – BOO loop (IF 2.32, 95%CI [1.45 to 3.18], *p* = .000), NTU – TTO – BOO loop (IF 1.94, 95%CI [1.20 to 2.68], *p* = .000) and NTU – TTO – FHO loop (IF 1.29, 95%CI [0.08 to 2.50], *p* = .037). Sensitivity analysis was conducted, and four studies were excluded from this network (study 5,9,16,22). Following this, no inconsistency was reported in the reconstructed network and the consistency model was adopted.

The BOO group had the shortest operation time (WMD compared with NTU −8.98, 95%CI [–14.07 to −3.88], SURCA 99.8%), followed by the TTO group (WMD −2.06, 95%CI [–3.29 to −0.82], SURCA 72.9%). No significant differences were found between other tourniquet application groups and the NTU group.

### 3.4. Blood loss

#### 3.4.1. Conventional pair-wise meta-analysis

Significant heterogeneity was found in intraoperative blood loss (*I*^2^ = 88.2%), postoperative blood loss (*I*^2^ = 59.2%) and total blood loss (*I*^2^ = 92.2%) so the random-effects model was adopted.

The use of a tourniquet overall was associated with a significantly decreased intraoperative blood loss compared to non-use of a tourniquet (WMD −142.86, 95% CI [–175.05 to −110.67]), but also with increased postoperative blood loss (WMD 81.28, 95% CI [35.23 to 127.33]). No significant difference was found in total blood loss (WMD −79.28, 95% CI [–177.34 to 18.78]).

#### 3.4.2. Network meta-analysis

Thirty-three articles were assessed in this network. No significant inconsistency was reported by the global inconsistency tests, node-split tests, or loop-specific inconsistency tests for the three blood loss indicators. The consistency model was more statistically suitable than the inconsistency model.

Apart from the MO group (WMD 47.21, 95% CI [–54.65 to 149.07]) and SHO group (WMD −34.63, 95%CI [–132.99 to 63.72]) which were not significantly different than NTU, other groups all demonstrated less intraoperative blood loss. The TTO group had the largest probability of having the least intraoperative blood loss (WMD −187.65, 95%CI [–254.33 to −120.97], SURCA 93.9%), followed by the BOO group (WMD −156.25, 95%CI [–260.93 to −51.56], SURCA 76.6%) and the FHO group (WMD −139.78, 95%CI [–212.47 to −67.08], SURCA 68.6%).

Only the TTO (WMD 84.95, 95% CI [1.47 to 168.43]) group showed more postoperative blood loss than the NTU group. No significant differences were found for any other groups compared with NTU. BOO (WMD −115.32, 95% CI [–195.56 to −35.08]) also was superior to TTU. The top three effective groups were BOO (SURCA = 84.1%), NTU (SURCA = 66.9%), and SHO (SURCA = 61.2%).

Similarly, the BOO (WMD −234.66, 95% CI [–409.19 to −60.13]) group and the TTO (WMD −116.63, 95% CI [–227.62 to −5.63]) group had less total blood loss than the NTU group. According to the SURCA value, the best strategy option was BOO (SUCRA = 92.8%), followed by SHO (SURCA = 64.7%) and TTO (SURCA = 60.2%). MO (SURCA = 66.9%) might be the least effective strategy other than NTU.

### 3.5. Pain

#### 3.5.1. Conventional pair-wise meta-analysis

Significant heterogeneity was found for both early (*I*^2^ = 97.3%) and late (*I*^2^ = 73.4%) postoperative pain so the random-effects model was adopted. No significant differences were found in early postoperative pain (SMD 0.23, 95% CI [–0.73 to 1.19]) or late postoperative pain (SMD −0.04, 95% CI [–0.34 to 0.27]).

#### 3.5.2. Network meta-analysis

Seventeen articles were assessed in this network. The consistency model was adopted because no significant inconsistency was reported by global inconsistency, node-split, or loop-specific inconsistency tests.

The SHO group demonstrated the most obvious early postoperative pain compared with the NTU group (SMD 1.10, 95% CI [0.13 to 2.08]) and any of the other tourniquet application groups. The BOO group had the greatest probability of being the best option (SUCRA = 96.5%), followed by SHO (SURCA = 61.8%) and TTO (SURCA = 53.9%).

No significant differences were found in the late postoperative pain network. BOO had the largest probability of being the best option (SMD −0.42, 95% CI [–1.25 to 0.42], SUCRA = 79.1%), followed by MO (SMD −0.21, 95% CI [–0.66 to 0.24], SURCA = 61.8%) and TTO (SMD −0.18, 95% CI [–0.55 to 0.20], SURCA = 63.2%). The greatest late postoperative pain was in the SHO group (SMD 0.14, 95% CI [–0.37 to 0.66], SURCA = 22.6%), but no significant difference was found compared with the NTU group.

### 3.6. Function

#### 3.6.1. Conventional pair-wise meta-analysis

Heterogeneity was found only in early postoperative function (*I*^2^ = 71.6%). The fixed-effects model was used for late postoperative function (*I*^2^ = 0.00%) and knee flexion range (*I*^2^ = 39.3%), and the random-effects model was adopted for early postoperative function. No significant difference was found in any functional outcome. The use or absence of tourniquets does not influence the patient’s early postoperative functional status (SMD −0.30, 95%CI [–0.682 to 0.073]), early postoperative functional status (SMD 0.05, 95%CI [–0.186 to 0.277]) or knee flexion range (WMD −0.79, 95%CI [–2.598 to 1.028]).

#### 3.6.2. Network meta-analysis

Seventeen articles with postoperative functional status and 15 articles with postoperative knee flexion range were assessed in this network. The consistency model was adopted for the early/late postoperative functional status because no inconsistency was reported in the global inconsistency, node-split, or loop-specific inconsistency tests. However, significant inconsistency was found in the postoperative knee flexion range. Based on the loop-specific inconsistency test, inconsistency was found in the TTO – MO – SHO loop (IF 25.39, 95%CI [21.53 to 29.25], *p* = .000) and the NTU – TTO – SHO loop (IF 20.82, 95%CI [12.92 to 28.71], *p* = .000). Sensitivity analysis was conducted, and one study was excluded from this network (study 37). Following this, no inconsistency was reported in the reconstructed network and the consistency model was adopted.

Similar to the results obtained by pair-wise meta-analysis, network meta-analysis did not find any significant differences in functional outcomes. According to the SURCA rank, theoretically, the best strategies for positive functional outcomes were SHO (knee flexion range, WMD 1.09, 95%CI [–2.07 to 4.25], SURCA = 85.4%), and FHO (early postoperative function, SMD 0.49, 95%CI [–0.82 to 1.80], SURCA = 89.1%; late postoperative function, SMD 0.37, 95%CI [–1.24 to 1.99], SURCA = 69.2%). The worst outcomes were TTO (knee flexion range, WMD −1.72, 95%CI [–3.93 to 0.50], SURCA = 20.9%), BOO (early postoperative function, SMD −0.99, 95%CI [–3.20 to 1.21], SURCA = 23.8%) and SHO (late postoperative function, SMD −0.45, 95%CI [–2.06 to 1.16], SURCA = 27.7%), respectively.

### 3.7. Safety outcome

#### 3.7.1. Conventional pair-wise meta-analysis

No heterogeneity was reported in safety outcomes (*I*^2^ = 0.00%) so the fixed-effects model was used. No significant differences were found between the use or absence of the tourniquet on the incidence of DVT (OR 1.39, 95%CI [0.879 to 2.209]), minor complications (OR 1.31, 95%CI [0.846 to 2.017]) or major complications (OR 0.95, 95%CI [0.251 to 3.555]).

#### 3.7.2. Network meta-analysis

Twenty-two articles were included in the safety network. The consistency model was adopted because no significant inconsistency was reported. Considering the actual clinical situation, the TTO group was chosen as the control group for this part of the analysis.

Consistent with expected results, the TTO group had a higher DVT incidence than any other group (the differences were all statistically significant). The SHO group had the lowest risk of postoperative DVT (OR −1.02, 95%CI [–2.52 to 0.47], SURCA = 72.2%) followed by FHO (OR −0.86, 95%CI [–1.66 to −0.06], SURCA = 71.3%) and NTU (OR −0.75, 95%CI [–1.31 to −0.19], SURCA = 64.9%).

Similar to the DVT results, the TTO group had a greater minor complication incidence than any other group (the differences were all statistically significant). The BOO group had the lowest risk of postoperative minor complications (OR-1.38, 95%CI [–3.00 to 0.25], SURCA = 83.9%) followed by MO (OR −1.00, 95%CI [–1.98 to −0.03], SURCA = 76.7%) and NTU (OR −0.68, 95%CI [–1.32 to −0.04], SURCA = 63.6%).

The TTO group had the highest risk for postoperative major complications (SURCA = 34.3%), but no significant difference was found when was compared with BOO (OR −0.09, 95%CI [–3.56 to 3.37]) or NTU (OR −0.12, 95%CI [–1.48 to 1.23]). The three strategies with lower risk were SHO (OR –1.03, 95%CI [–2.90 to 0.84], SURCA = 77.4%), MO (OR −0.37, 95%CI [–1.58 to 0.84], SURCA = 55.5%) and FHO (OR –0.19, 95%CI [–1.37 to 1.00], SURCA = 45.7%).

## 4. Discussion

This is the first study that comprehensively compared different tourniquet application strategies to evaluate their impact on postoperative recovery following TKA.

Previous systematic reviews have indicated that the use of tourniquets can reduce surgical bleeding to a certain extent, but can cause vascular endothelial injury or ischemia-reperfusion injury of the lower limbs, resulting in poor wound healing, DVT, swelling, increased postoperative pain, and loss of function.

To comprehensively weigh the pros and cons of tourniquet use to help clinicians make decisions, five clinically important endpoints were assessed in this study: perioperative blood loss, operation time, postoperative pain and function, and complications. All relevant RCTs were screened and 38 eligible RCTs were included in the study. A frequentist framework network meta-analysis was conducted. This method integrates all available direct or indirect evidence from RCTs comparing different tourniquet application strategies, thereby augmenting the number of studies within each comparison, which increases the power of the study. For instance, only five of the included studies compared SHO with other strategies, but through the network, there were 38 studies allowing six indirect comparisons.

The main results of this analysis revealed that: (1) BOO and TTO result in shorter operation times. Probably for this reason they also had less intraoperative and total blood loss; (2) TTO had more postoperative blood loss, while BOO had the least postoperative bleeding; (3) SHO may cause more early postoperative pain, but no difference was found in late postoperative pain; (4) BOO had the lowest early and late postoperative pain, but could negatively affect early functional recovery following TKA; (5) TTO was related to a greater incidence of DVT, as well as minor and major complications. BOO had the lowest risk of postoperative minor complications, whereas SHO had the lowest risk of postoperative DVT and major complications; (6) Regardless of whether a tourniquet is used or how it is used, the late knee pain and function following TKA will not be affected; (7) According to the results of the cluster-rank analysis, it is safer and more effective to use the BOO strategy to prevent DVT and to reduce bleeding and minor complications simultaneously, but SHO is more suitable for patients who are at high risk of major complications.

Alcelik I et al. [15] supposed that using a tourniquet to decrease blood loss may involve two main effects. First, bleeding at the surgical site might be reduced, consequently reducing blood loss during surgery. Second, if there is little bleeding from the wound, the surgeon could spend less time controlling the bleeding, so the operation time should be shortened. According to our results, TTO and BOO were associated with shorter operation time and less intraoperative and total blood loss. This is in line with Alcelik I’s speculation, but we believe that shortening the operation time as much as possible and closing the wound in advance also has a positive effect on reducing intraoperative blood loss. Theoretically, the use of a tourniquet may cause calcification of the vascular endothelium, blood flow stasis of the lower limbs, and ischemia-reperfusion injury to potentially increase the risk of postoperative complications. Previous studies have proven that a higher rate of complications was related to longer tourniquet times [57–59]. Olivecrona C et al. found that every additional 10 min of tourniquet time was associated with an increased risk for complications [60]. Because using a tourniquet throughout the surgery can reduce blood loss effectively, it is indeed related to many safety issues. Thus, it is crucial to find a balance between reducing surgical bleeding and shortening surgical time, i.e. the best tourniquet application strategy. According to our results, tourniquet inflation before osteotomy then deflation after wound closure may be the ideal choice, with the lowest perioperative blood loss and consequently the shortest operation time and the lowest risk of complications.

There are several limitations to this study. First, there are some inevitable confounding factors that may affect the stability of the results. For instance, the skills of the surgeons will undoubtedly affect the operation time and blood loss. It is an uncontrollable variable when comparing these studies and is impossible to control or eliminate by statistical means. Second, considering the unmanageable confounding factors in observational studies, only RCTs were included even though observational studies also can play an important role in evaluating the effectiveness and safety of tourniquet application following TKA. This may contribute to the small number of studies we could include. Although funnel plots and Egger’s tests were performed in this study and no significant publication bias was found, bias can be a potential problem especially when the funnel plots showed a dubious asymmetry. Finally, the DVT and major complication networks inevitably involved rare event rates. Although the Cochrane Handbook recommends the omission of studies with no events in both arms, it is still controversial whether this exclusion reduces the coverage rates of confidence intervals and increases the bias estimates. Previous studies have shown that including such trials produces unbiased estimation with narrow confidence intervals, improving the accuracy of combined estimation in a meta-analysis. The results of this should be interpreted cautiously. More high-quality trials are needed.

## 5. Conclusion

Using tourniquets during the entire operation can effectively reduce blood loss, but it also can cause many safety problems, including DVTs and wound complications. Tourniquet inflation before osteotomy then deflation after wound closure effectively can reduce perioperative bleeding while shortening the operation time and reducing postoperative complications. We conclude that tourniquet inflation before osteotomy then deflation after wound closure could be the ideal tourniquet application strategy in TKA.

## Supplementary Material

Supplemental Material

## Data Availability

The authors confirm that the data supporting the findings of this study are available within the article and its supplementary materials.

## Abbreviations

**Abbreviations**
BOO: before osteotomy in operation
CI: confidence intervals
DVT: deep vein thrombosis
FHO: first half of operation
IFs: inconsistency factors
IQR: interquartile range
KOA: knee osteoarthritis
MO: middle of operation
NTU: non-use of a tourniquet
OR: odds ratio
PRISMA: Preferred Reporting Items for Systematic Reviews and Meta-Analyses guidelines
RA: rheumatoid arthritis
SMD: standardized mean difference
SHO: second half of operation
SUCRA: surface under the cumulative ranking
TKA: total knee arthroplasty
TTO: throughout the operation
WMD: weighted mean difference
WOMAC: Western Ontario and McMaster Universities Arthritis Index
