# Supplementary material for: Comparison of efficacy and safety of different tourniquet applications in total knee arthroplasty: a network meta-analysis of randomized controlled trials
Source: Ann Med. 2021 Nov 2;53(1):1816–26. doi: 10.1080/07853890.2021.1991588 (PMC8567921; doi:10.1080/07853890.2021.1991588)
Supplement: Supplemental Material [file IANN_A_1991588_SM8059.zip › Supplementary_appendix_documents.docx]

**Supplementary figure 1.** PRISMA Flow Diagram.

**Supplementary figure 2.** Publication bias and Egger test for main networks. (A) Operation time. (B) Intraoperative blood loss. (C) postoperative blood loss. (D) Total blood loss. (E) Knee flexion range. (F) Early postoperative function. (G) Late postoperative function. (H) Early postoperative pain. (I) Late postoperative pain. (J) DVT. (K) Minor complications. (L) Major complications.

**Supplementary figure 3.** Cluset-rank plots. (A) The cluster-rank plot of total blood loss and DVT. (B) The cluster-rank plot of total blood loss and operation time. (C) The cluster-rank plot of total blood loss and minor complications. (D) The cluster-rank plot of total blood loss and major complications. (E) The cluster-rank plot of total blood loss and early postoperative pain. (F) The cluster-rank plot of total blood loss and late postoperative pain. (G) The cluster-rank plot of total blood loss and early postoperative function. (H) The cluster-rank plot of total blood loss and late postoperative function. (I) The cluster-rank plot of total blood loss and knee flexion range.(The cluster-rank value is the product of the abscissa and ordinate of each treatment).

**Supplementary table 1.** Baseline Characteristics of included Studies

**Supplementary table 2**. Methodological quality and risk of bias evaluation. L: low risk of bias. U: unclear risk of bias. H: high risk of bias.

**Supplementary table 3**. Detailed results of network meta-analysis of blood loss and operation time.

**Supplementary table 4**. Detailed results of network meta-analysis of postoperative pain and function.

**Supplementary table 5**. Detailed results of network meta-analysis of safety outcomes.


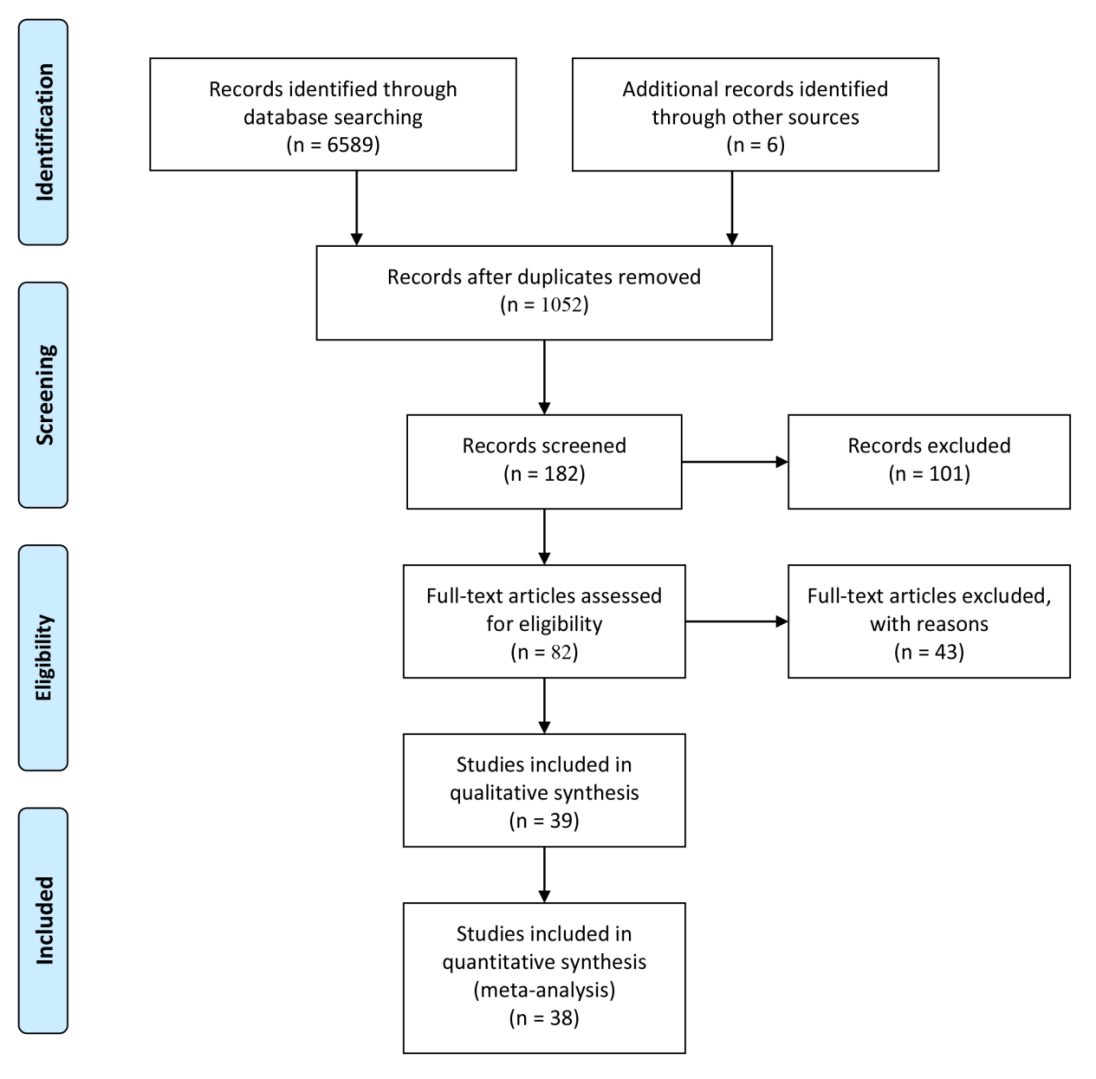


**Supplementary figure 1.** PRISMA Flow Diagram.


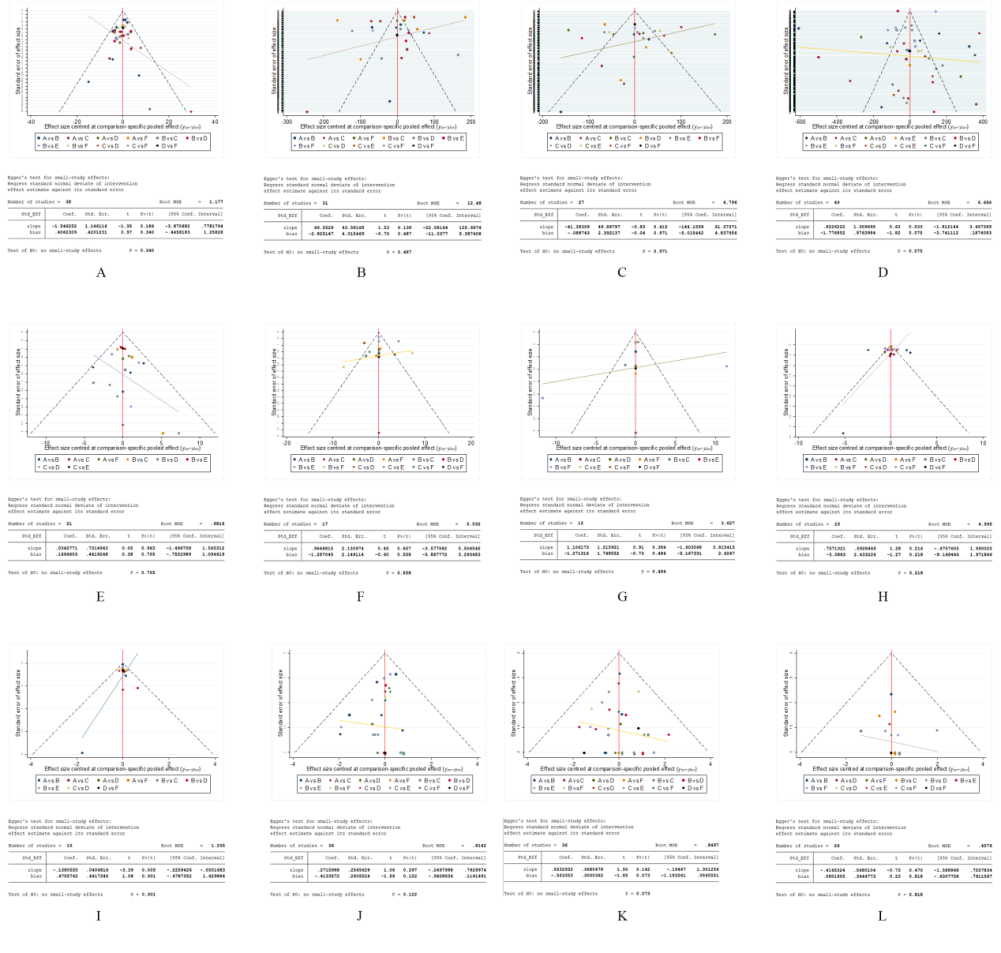


**Supplementary figure 2.** Publication bias and results of Egger tests. (A) Operation time. (B) Intraoperative blood loss. (C) postoperative blood loss. (D) Total blood loss. (E) Knee flexion range. (F) Early postoperative function. (G) Late postoperative function. (H) Early postoperative pain. (I) Late postoperative pain. (J) DVT. (K) Minor complications. (L) Major complications.


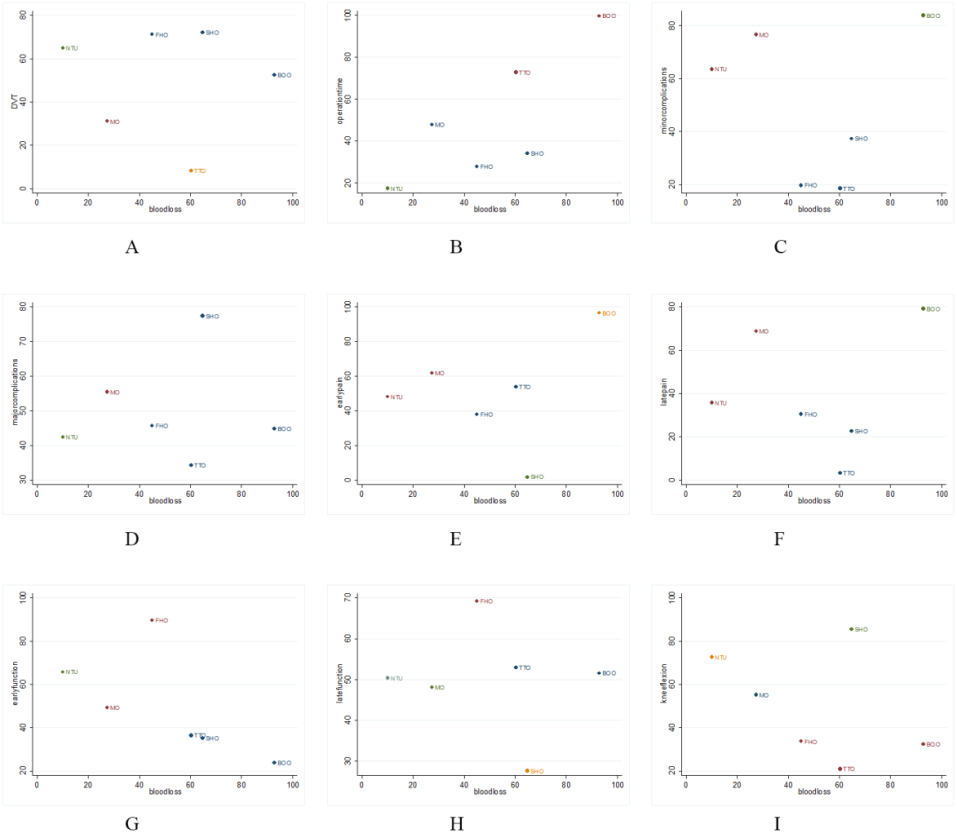


**Supplementary figure 3.** Cluset-rank plots. (A) The cluster-rank plot of total blood loss and DVT. (B) The cluster-rank plot of total blood loss and operation time. (C) The cluster-rank plot of total blood loss and minor complications. (D) The cluster-rank plot of total blood loss and major complications. (E) The cluster-rank plot of total blood loss and early postoperative pain. (F) The cluster-rank plot of total blood loss and late postoperative pain. (G) The cluster-rank plot of total blood loss and early postoperative function. (H) The cluster-rank plot of total blood loss and late postoperative function. (I) The cluster-rank plot of total blood loss and knee flexion range.(The cluster-rank value is the product of the abscissa and ordinate of each treatment).

**Supplementary table 1.** Baseline Characteristics of included Studies

| Author | No. | Year | Number of patients | Mean age | Male/Female | Diagnosis | Surgical approach | The use of drainage (Yes/No) | The use of tranexamic acid (Yes/No) | The use of anticoagulant (Yes/No,category) | Control intervention I | Control intervention II | Control intervention III |
| --- | --- | --- | --- | --- | --- | --- | --- | --- | --- | --- | --- | --- | --- |
| Kvederas G, et al[11]. | 1 | 2012 | 36 | 69.47 | 5/31 | Osteoarthritis | Medial subvastus approach | Yes | No | Yes/LMWH | Tourniquet inflation before incision and deflation after cement hardening. | Tourniquet inflating merely during cementing. | Tourniquet inflation before incision and deflation after wound closure. |
| Hasanain MS et al [24]. | 2 | 2018 | 54 | 62.93 | 20/34 | Osteoarthritis, Rheumatoid arthritis | Subvastus approach | Yes | No | Yes/DTI | Tourniquet inflation before incision and deflation after wound closure. | Tourniquet inflating merely during cementing. | / |
| Huang ZY et al [25]. | 3 | 2014 | 90 | 66.2 | 31/59 | Osteoarthritis | Mini-midvastus approach | Yes | No | Yes/LMWH | Tourniquet inflation before incision and deflation after wound closure. | Tourniquet inflation before incision and deflation after cement hardening. | Tourniquet inflating merely during cementing. |
| Rathod P et al [13]. | 4 | 2014 | 80 | 63.85 | 37/43 | Osteoarthritis | Medial parapatellar approach | No | No | Yes/AntiK | Tourniquet inflation before incision and deflation after wound closure. | Tourniquet inflating merely during cementing. | / |
| Ishii Y et al [26]. | 5 | 2005 | 55 | 71 | 6/49 | Osteoarthritis, Rheumatoid arthritis | NR | Yes | No | No | Tourniquet inflation before incision and deflation after cement hardening. | Tourniquet inflation before incision and deflation after wound closure. | / |
| Fukuda A et al [27]. | 6 | 2007 | 48 | 70.23 | 7/41 | Osteoarthritis | Midvastus approach | Yes | No | No | No tourniquet was used | Tourniquet inflation before incision and deflation after cement hardening. | / |
| Aglietti P et al [28]. | 7 | 2000 | 20 | 69 | 7/13 | Osteoarthritis | Medial parapatellar approach | Yes | No | No | Tourniquet inflation before incision and deflation after cement hardening. | No tourniquet was used | / |
| Harvey EJ et al [29]. | 8 | 1997 | 80 | 70.91 | NA | Osteoarthritis, Rheumatoid arthritis, Revision | Medial parapatellar approach | Yes | No | Yes/AntiK/LMWH | No tourniquet was used | Tourniquet inflating merely during cementing. | Tourniquet inflation before incision and deflation after wound closure. |
| Zhang Y et al [30]. | 9 | 2017 | 150 | 69.7 | 68/82 | Osteoarthritis | Medial parapatellar approach | Yes | No | Yes/LMWH | Tourniquet inflation before incision and deflation after wound closure. | Tourniquet inflation before incision and deflation after cement hardening. | Tourniquet inflation before osteotomy and deflation after wound closure. |
| Tai TW et al [12]. | 10 | 2012 | 72 | 71.8 | 17/55 | Osteoarthritis | Medial parapatellar approach | Yes | No | No | Tourniquet inflation before incision and deflation after wound closure. | No tourniquet was used | / |
| Ejaz A et al [14]. | 11 | 2014 | 64 | 68 | 35/29 | Osteoarthritis | Medial parapatellar approach | Yes | Yes | Yes/DTI | Tourniquet inflation before incision and deflation after wound closure. | No tourniquet was used | / |
| Kageyama K et al [31]. | 12 | 2007 | 22 | 74.5 | 4/18 | Osteoarthritis | NR | Yes | No | No | No tourniquet was used | Tourniquet inflation before incision and deflation after wound closure. | / |
| Zhou K et al [32]. | 13 | 2017 | 140 | 67.92 | 20/120 | Osteoarthritis, Rheumatoid arthritis | Medial parapatellar approach | Yes | Yes | Yes/DTI | No tourniquet was used | Tourniquet inflation before incision and deflation after wound closure. | / |
| Hernández-Castaños DM et al [33]. | 14 | 2008 | 43 | 75.95 | 13/30 | Osteoarthritis | Medial parapatellar approach | Yes | No | Yes/LMWH | Tourniquet inflation before incision and deflation after cement hardening. | Tourniquet inflation before incision and deflation after wound closure. | / |
| Hakkalamani S et al [34]. | 15 | 2015 | 60 | 67.85 | 30/30 | Osteoarthritis | Medial parapatellar approach | No | No | Yes/DTI | Tourniquet inflation before incision and deflation after wound closure. | Tourniquet inflating merely during cementing. | / |
| Widman J et al [35]. | 16 | 1999 | 85 | 71.54 | 22/63 | Osteoarthritis, Rheumatoid arthritis | Medial parapatellar approach | Yes | No | No | Tourniquet inflation before incision and deflation after cement hardening. | Tourniquet inflation before incision and deflation after wound closure. | / |
| Vandenbussche E et al [36]. | 17 | 2002 | 80 | 70.5 | 25/55 | Osteoarthritis | NR | Yes | No | Yes/LMWH | Tourniquet inflation before incision and deflation after wound closure. | No tourniquet was used | / |
| Li B et al [37]. | 18 | 2009 | 80 | 70.5 | 24/56 | Osteoarthritis, Rheumatoid arthritis | Midvastus approach | Yes | No | Yes/LMWH | Tourniquet inflation before incision and deflation after wound closure. | No tourniquet was used | / |
| Tetro AM et al [38]. | 19 | 2001 | 63 | 69.8 | 26/37 | Osteoarthritis, Rheumatoid arthritis | NR | Yes | No | NR | Tourniquet inflation before incision and deflation after cement hardening. | No tourniquet was used | / |
| Wang K et al [39]. | 20 | 2016 | 50 | 72.4 | 9/41 | Osteoarthritis, Rheumatoid arthritis | Medial parapatellar approach | Yes | No | Yes/DTI | Tourniquet inflation before incision and deflation after cement hardening. | Tourniquet inflating merely during cementing. | / |
| Chen S et al [40]. | 21 | 2014 | 64 | 71.4 | 17/47 | Osteoarthritis, Rheumatoid arthritis | Medial parapatellar approach | Yes | No | Yes/DTI | Tourniquet inflation before osteotomy and deflation after wound closure. | Tourniquet inflation before incision and deflation after wound closure. | / |
| Yavarikia A et al [41]. | 22 | 2010 | 84 | 65.74 | 22/62 | Osteoarthritis | NR | Yes | No | Yes/LMWH | No tourniquet was used | Tourniquet inflation before incision and deflation after cement hardening. | Tourniquet inflation before osteotomy and deflation after wound closure. |
| Fan Y et al [57]. | 23 | 2014 | 60 | 64.32 | 16/44 | Osteoarthritis, Rheumatoid arthritis | Mini medial parapatellar approach | Yes | No | Yes/LMWH | Tourniquet inflation before incision and deflation after wound closure. | Tourniquet inflation before osteotomy and deflation after wound closure. | / |
| Tarwala R et al [56]. | 24 | 2013 | 71 | 65.34 | 27/44 | Osteoarthritis | Medial parapatellar approach | Yes | Yes | Yes/AntiP | Tourniquet inflation before incision and deflation after wound closure. | Tourniquet inflating merely during cementing. | / |
| Hersekli MA et al [42]. | 25 | 2004 | 76 | 66.58 | 10/66 | Osteoarthritis | Medial parapatellar approach | Yes | No | Yes/AntiK | Tourniquet inflation before incision and deflation after cement hardening. | Tourniquet inflation before incision and deflation after wound closure. | / |
| Dennis DA et al [43]. | 26 | 2015 | 28 | 62 | 16/12 | Osteoarthritis | Medial parapatellar approach | Yes | No | Yes | Tourniquet inflation before incision and deflation after cement hardening. | No tourniquet was used | / |
| Yin D et al [44]. | 27 | 2017 | 80 | 64.55 | 30/48 | Osteoarthritis | Medial parapatellar approach | Yes | No | Yes/LMWH | Tourniquet inflation before incision and deflation after wound closure. | Tourniquet inflating merely during cementing. | / |
| Ejaz A et al [45]. | 28 | 2015 | 62 | 68.25 | 33/29 | Osteoarthritis | Medial parapatellar approach | Yes | No | NR | Tourniquet inflation before incision and deflation after wound closure. | No tourniquet was used |  |
| Goel R et al [46]. | 29 | 2019 | 200 | 65.75 | 100/100 | Osteoarthritis | Medial parapatellar approach | Yes | No | Yes/AntiK | Tourniquet inflation before incision and deflation after wound closure. | No tourniquet was used | / |
| Mori N et al [47]. | 30 | 2015 | 103 | 73.71 | 15/88 | Osteoarthritis | Midvastus approach | NR | NR | NR | Tourniquet inflation before incision and deflation after wound closure. | No tourniquet was used | / |
| Schnettler T et al [48]. | 31 | 2017 | 81 | 65.09 | 26/55 | Osteoarthritis, Rheumatoid arthritis | NR | Yes | Yes | Yes/AntiK | Tourniquet inflating merely during cementing. | No tourniquet was used | / |
| Huiling G et al [49]. | 32 | 2013 | 46 | 64.06 | 14/32 | Osteoarthritis | Medial parapatellar approach | Yes | No | NR | Tourniquet inflation after osteotomy and deflation after wound closure. | Tourniquet inflation before incision and deflation after wound closure. | / |
| Mengjian Z et al [50]. | 33 | 2016 | 90 | 65.4 | 19/71 | Osteoarthritis | Medial parapatellar approach | Yes | Yes | Yes/DTI | Tourniquet inflation before incision and deflation after wound closure. | Tourniquet inflation before incision and deflation after cement hardening. | Tourniquet inflation after osteotomy and deflation after wound closure. |
| Xinling W et al [51]. | 34 | 2019 | 90 | 63.47 | 17/73 | Osteoarthritis | Medial parapatellar approach | Yes | Yes | Yes/LMWH | Tourniquet inflating merely during cementing. | Tourniquet inflation after osteotomy and deflation after wound closure. | No tourniquet was used |
| Jun F et al [52]. | 35 | 2016 | 106 | 65 | 47/59 | Osteoarthritis | NR | Yes | No | Yes/DTI | Tourniquet inflation before osteotomy and deflation after wound closure. | Tourniquet inflation before incision and deflation after wound closure. | / |
| Weishan L et al [53]. | 36 | 2018 | 150 | 60.2 | 78/72 | Osteoarthritis | Medial parapatellar approach | Yes | No | Yes/LMWH | Tourniquet inflation before incision and deflation after wound closure. | Tourniquet inflation before incision and deflation after cement hardening. | Tourniquet inflation before osteotomy and deflation after wound closure. |
| Qiang Z et al [54]. | 37 | 2017 | 78 | 60.2 | 38/40 | Osteoarthritis | Medial parapatellar approach | Yes | No | Yes/LMWH | Tourniquet inflation before incision and deflation after wound closure. | Tourniquet inflating merely during cementing. | Tourniquet inflation after osteotomy and deflation after wound closure. |
| Qi Z et al [55]. | 38 | 2016 | 166 | 64.2 | 25/141 | Osteoarthritis | Medial parapatellar approach | Yes | No | Yes/DTI | Tourniquet inflation after osteotomy and deflation after wound closure. | No tourniquet was used | / |

**Supplementary table 2.** Methodological quality and risk of bias evaluation. L: low risk of bias. U: unclear risk of bias. H: high risk of bias.

| Author | No. | 1.Sequence generation | 2.Allocation concealment | 3.Blinding | 4.Incomplete outcome data | 5.Selective outcome reporting | 6.Other source of bias |
| --- | --- | --- | --- | --- | --- | --- | --- |
| Kvederas G, et al[11]. | 1 | L | L | U | H | L | L |
| Hasanain MS et al [24]. | 2 | U | L | L | L | L | L |
| Huang ZY et al [25]. | 3 | U | L | L | L | L | H |
| Rathod P et al [13]. | 4 | U | L | L | L | L | L |
| Ishii Y et al [26]. | 5 | U | L | U | L | L | L |
| Fukuda A et al [27]. | 6 | U | L | U | L | L | L |
| Aglietti P et al [28]. | 7 | U | L | L | L | L | L |
| Harvey EJ et al [29]. | 8 | U | L | L | L | L | L |
| Zhang Y et al [30]. | 9 | L | U | L | L | L | L |
| Tai TW et al [12]. | 10 | U | L | L | L | L | L |
| Ejaz A et al [14]. | 11 | U | L | U | L | L | L |
| Kageyama K et al [31]. | 12 | L | L | L | L | L | U |
| Zhou K et al [32]. | 13 | L | L | H | L | L | L |
| Hernández-Castaños DM et al [33]. | 14 | U | L | U | L | L | L |
| Hakkalamani S et al [34]. | 15 | L | L | L | L | L | U |
| Widman J et al [35]. | 16 | L | U | L | L | L | U |
| Vandenbussche E et al [36]. | 17 | L | U | L | L | L | H |
| Li B et al [37]. | 18 | L | U | L | L | L | U |
| Tetro AM et al [38]. | 19 | L | U | L | L | L | U |
| Wang K et al [39]. | 20 | L | L | L | L | L | U |
| Chen S et al [40]. | 21 | L | L | U | L | L | U |
| Yavarikia A et al [41]. | 22 | L | L | U | L | L | L |
| Fan Y et al [57]. | 23 | L | L | L | L | L | U |
| Tarwala R et al [56]. | 24 | L | L | L | L | L | U |
| Hersekli MA et al [42]. | 25 | L | L | L | L | L | U |
| Dennis DA et al [43]. | 26 | L | L | L | L | L | U |
| Yin D et al [44]. | 27 | L | L | L | L | L | U |
| Ejaz A et al [45]. | 28 | L | L | L | L | L | U |
| Goel R et al [46]. | 29 | L | L | L | L | L | U |
| Mori N et al [47]. | 30 | L | L | L | L | L | U |
| Schnettler T et al [48]. | 31 | U | L | L | L | L | U |
| Huiling G et al [49]. | 32 | U | L | U | L | L | L |
| Mengjian Z et al [50]. | 33 | L | U | U | L | L | L |
| Xinling W et al [51]. | 34 | L | U | U | L | L | L |
| Jun F et al [52]. | 35 | L | U | U | L | L | L |
| Weishan L et al [53]. | 36 | L | U | U | L | L | L |
| Qiang Z et al [54]. | 37 | L | U | U | L | L | L |
| Qi Z et al [55]. | 38 | L | H | U | L | L | L |

**Supplementary table 3**. Detailed results of network meta-analysis of blood loss and operation time.

| Treatment | WMD (95%CI)  for Operation time | SURCA for Operation time, % | WMD (95%CI)  for Intraoperative blood loss | SURCA  for Intraoperative blood loss, % | WMD (95%CI)  for Postoperative blood loss | SURCA  for Postoperative blood loss, % | WMD (95%CI)  for Total blood loss | SURCA  for Total blood loss, % |
| --- | --- | --- | --- | --- | --- | --- | --- | --- |
| NTU | Reference | 17.5 | Reference | 21.5 | Reference | 66.9 | Reference | 10.0 |
| TTO | -8.98 (-14.07 to -3.88) | 72.9 | -187.65 (-254.33 to -120.97) | 93.3 | 84.95 (1.47 to 168.43) | 5.4 | -116.63 (-227.62 to -5.63) | 60.2 |
| FHO | -2.06 (-3.29 to -0.82) | 27.8 | -139.78 (-212.47 to -67.08) | 68.6 | 49.09 (-34.40 to 132.58) | 29.4 | -86.18 (-213.33 to 40.97) | 45.0 |
| MO | -1.10 (-3.39 to 1.18) | 47.9 | 47.21 (-54.65 to 149.07) | 5.2 | 20.52 (-90.23 to 131.27) | 53.0 | -45.63 (-194.29 to 103.03) | 27.3 |
| BOO | -0.56 (-2.92 to 1.79) | 99.8 | -156.25 (-260.93 to -51.56) | 76.6 | -30.37 (-141.17 to 80.43) | 84.1 | -234.66 (-409.19 to -60.13) | 92.8 |
| SHO | -0.35 (-2.70 to 2.01) | 34.1 | -34.63 (-132.99 to 63.72) | 34.3 | 7.66 (-111.44 to 126.77) | 61.2 | -141.40 (-371.14 to 88.34) | 64.7 |

**Supplementary table 4**. Detailed results of network meta-analysis of postoperative pain and function.

| Treatment | WMD (95%CI)  for Knee flexion range | SURCA  for Knee flexion range, % | SMD (95%CI)  for Early postoperative function | SURCA  for Early postoperative function, % | SMD (95%CI)  for Late postoperative function | SURCA  for Late postoperative function, % | SMD (95%CI)  for Early postoperative pain | SURCA  for Early postoperative pain, % | SMD (95%CI)  for Late postoperative pain | SURCA  for Late postoperative pain, % |
| --- | --- | --- | --- | --- | --- | --- | --- | --- | --- | --- |
| NTU | Reference | 72.7 | Reference | 65.7 | Reference | 50.4 | Reference | 48.1 | Reference | 35.7 |
| TTO | -1.72 (-3.93 to 0.50) | 20.9 | -0.52 (-1.80 to 0.76) | 36.4 | 0.06 (-1.47 to 1.59) | 53.0 | -0.08 (-0.79 to 0.62) | 53.9 | -0.18 (-0.55 to 0.20) | 63.2 |
| FHO | -1.45 (-4.07 to 1.17) | 33.6 | 0.49 (-0.82 to 1.80) | 89.7 | 0.37 (-1.24 to 1.99) | 69.2 | 0.14 (-0.83 to 1.11) | 38.0 | 0.05 (-0.51 to 0.62) | 30.5 |
| MO | -0.87 (-3.67 to 1.92) | 55.2 | -0.28 (-1.73 to 1.18) | 49.3 | -0.04 (-2.18 to 2.11) | 48.1 | -0.18 (-1.04 to 0.68) | 61.8 | -0.21 (-0.66 to 0.24) | 68.9 |
| BOO | -1.51 (-4.06 to 1.03) | 32.3 | -0.99 (-3.20 to 1.21) | 23.8 | 0.04 (-2.38 to 2.46) | 51.6 | -1.10 (-2.32 to 0.12) | 96.5 | -0.42 (-1.25 to 0.42) | 79.1 |
| SHO | 1.09 (-2.07 to 4.25) | 85.4 | -0.54 (-1.78 to 0.71) | 35.1 | -0.45 (-2.06 to 1.16) | 27.7 | 1.10 (0.13 to 2.08) | 1.7 | 0.14 (-0.37 to 0.66) | 22.6 |

**Supplementary table 5**. Detailed results of network meta-analysis of safety outcomes.

| Treatment | OR (95%CI)  for Incidence of DVT | SURCA  for Incidence of DVT, % | OR (95%CI)  for Minor complication | SURCA  for Minor complication, % | OR (95%CI)  for Major complication | SURCA  for Major complication, % |
| --- | --- | --- | --- | --- | --- | --- |
| NTU | -0.75 (-1.31 to -0.19) | 64.9 | -0.68 (-1.32 to -0.04) | 63.6 | -0.12 (-1.48 to 1.23) | 42.3 |
| TTO | Reference | 8.2 | Reference | 18.6 | Reference | 34.3 |
| FHO | -0.86 (-1.66 to -0.06) | 71.3 | -0.00 (-0.75 to 0.75) | 19.8 | -0.19 (-1.37 to 1.00) | 45.7 |
| MO | -0.28 (-1.18 to 0.61) | 31.1 | -1.00 (-1.98 to -0.03) | 76.7 | -0.37 (-1.58 to 0.84) | 55.5 |
| BOO | -0.58 (-1.19 to 0.03) | 52.4 | -1.38 (-3.00 to 0.25) | 83.9 | -0.09 (-3.56 to 3.37) | 44.8 |
| SHO | -1.02 (-2.52 to 0.47) | 72.2 | -0.26 (-1.40 to 0.89) | 37.4 | -1.03 (-2.90 to 0.84) | 77.4 |
