# Supplementary figures and images for: Comparison of efficacy and safety of different tourniquet applications in total knee arthroplasty: a network meta-analysis of randomized controlled trials
Source: Ann Med. 2021 Nov 2;53(1):1816–26. doi: 10.1080/07853890.2021.1991588 (PMC8567921; doi:10.1080/07853890.2021.1991588)

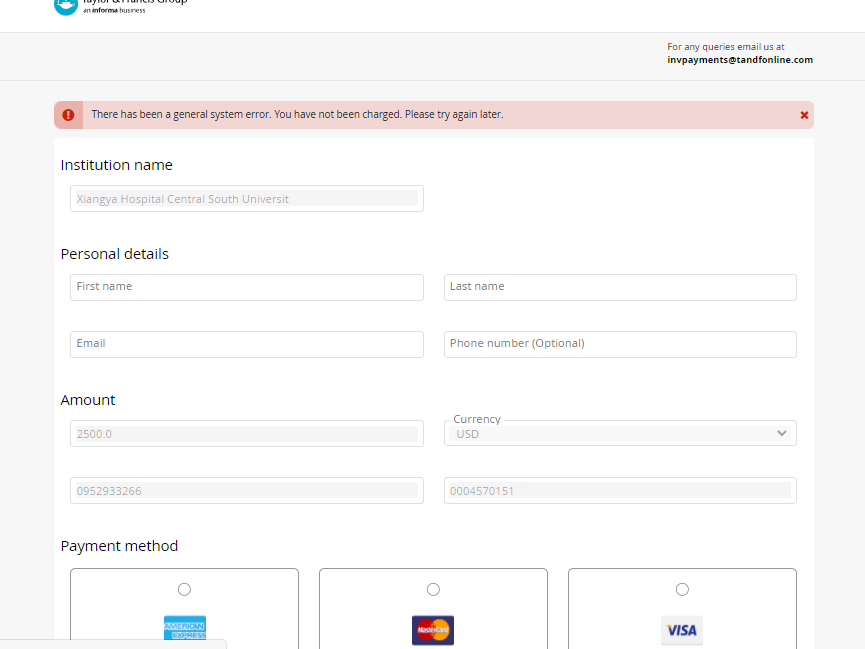

Supplement: Supplemental Material [file IANN_A_1991588_SM8059.zip › __20211025233240.png]
